# Supplementary material for: Housing Systems Affect Eggshell Lightness and Free Amino Acid Contents of Egg Albumen in Tosa-Jidori Chickens: A Preliminary Research
Source: Animals (Basel). 2023 Jun 1;13(11):1837. doi: 10.3390/ani13111837 (PMC10251838; doi:10.3390/ani13111837)
Supplement: Supplementary file 1 [file animals-13-01837-s001.zip › animals-2364265-supplementary.pdf]

Housing systems affect eggshell lightness and free amino acid contents of egg albumen in Tosa-jidori chickens: a preliminary research

Nonoka Kawamura, Masahiro Takaya, Hideaki Hayashi, Tatsuhiko Goto

Supplementary Materials

Figure S1. Egg production rates (%) in cage and litter hens.
Egg production rates (%) during 1 week in cage and litter are calculated through the experimental period (from 0 wk to 19 wk). Data are shown as the mean ± SE. There are no significant differences between cage and litter in each stage (0-19 wks) ($p > 0.05$), except for 3 wk ($p < 0.05$).

Figure S2. Phenotypic correlations of cage hens in first stage.
Egg traits, yolk amino acids traits, albumen amino acids traits, and body weight gain from cage hens in the first stage are used. Trait abbreviations are indicated in the main text. Pearson's correlations are expressed by ellipses. The Blue ellipse indicates positive correlations whereas the red indicates negative correlations in each pair ($p < 0.05$). Blank means no correlations.

Figure S3. Phenotypic correlations of litter hens in first stage.
Egg traits, yolk amino acids traits, and albumen amino acids traits from litter floor hens in the first stage are used. Trait abbreviations are indicated in the main text. Pearson's correlations are expressed by ellipses. The Blue ellipse indicates positive correlations whereas the red indicates negative correlations in each pair ($p < 0.05$). Blank means no correlations.

Figure S4. Phenotypic correlations of litter hens in second stage.
Egg traits, yolk amino acids traits, and albumen amino acids traits from litter floor hens in the second stage are used. Trait abbreviations are indicated in the main text. Pearson's correlations are expressed by ellipses. The Blue ellipse indicates positive correlations whereas the red indicates negative correlations in each pair ($p < 0.05$). Blank means no correlations.

Table S1. Egg index traits measured in first and second stages in two housing systems.

Traits	Cage			Litter			One-way ANOVA				
							df_{bet}	df_{res}	F value	P value	
First stage	(n = 20)			(n = 20)							
Egg shape index	75.7	±	2.1	76.9	±	2.6	1	38	2.705	0.118	
Yolk weight (%)	29.5	±	1.5	29.3	±	2.3	1	38	0.038	0.847	
Eggshell weight (%)	14.4	±	1.6	13.3	±	1.3	1	38	5.402	0.026	*
Albumen weight (%)	56.1	±	2.4	57.3	±	1.9	1	38	2.912	0.096	
Second stage	(n = 20)			(n = 19)							
Egg shape index	76.0	±	2.4	77.5	±	2.1	1	37	3.892	0.056	
Yolk weight (%)	30.8	±	2.0	29.6	±	1.2	1	37	4.772	0.035	*
Eggshell weight (%)	13.8	±	0.9	13.5	±	0.9	1	37	0.789	0.380	
Albumen weight (%)	55.4	±	2.2	56.8	±	1.4	1	37	5.669	0.023	*

The egg shape index is the ratio of the short axis to the long axis of the egg. The percent of yolk, eggshell, and albumen are based on egg weight. *$p < 0.05$. Mean ± SD. df_{bet}: between groups degree of freedom. df_{res}: residual degree of freedom.

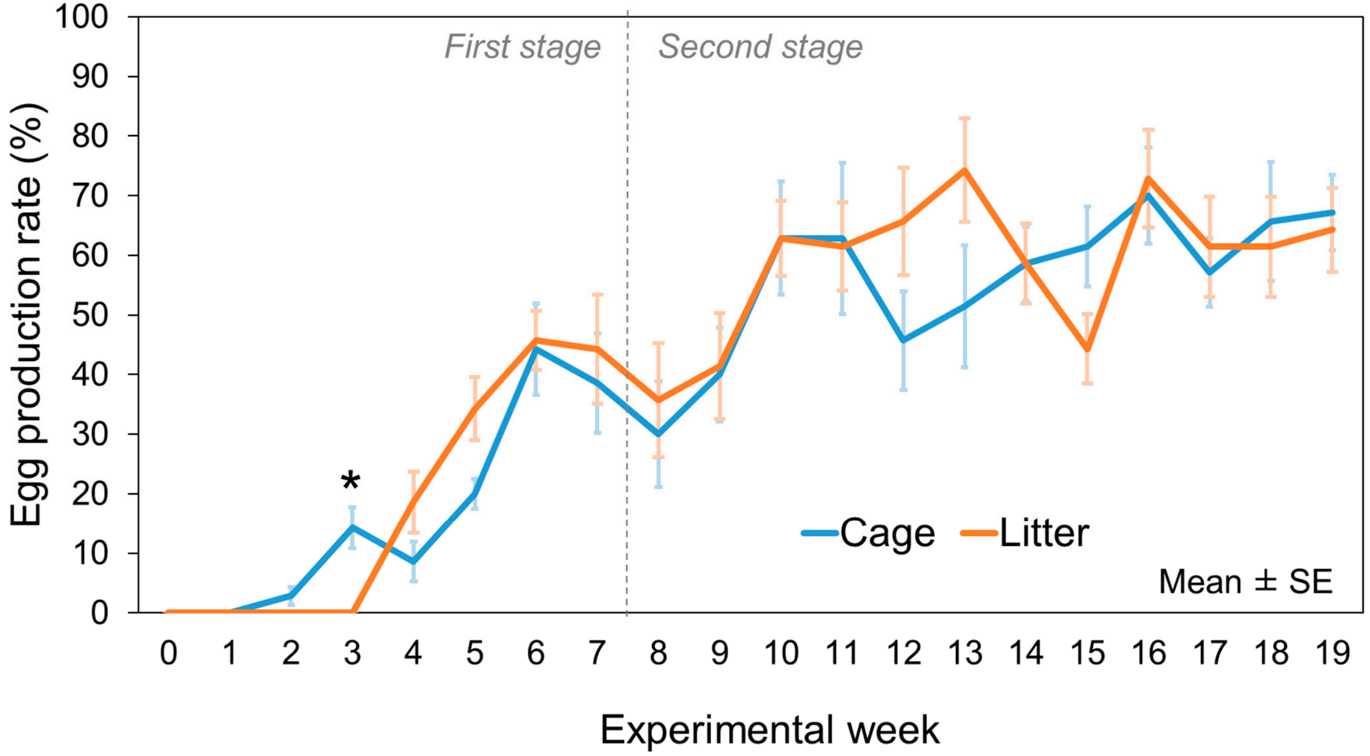
First stage
Second stage
Egg production rate (%)
100
90
80
70
60
50
40
30
20
10
0
*
Cage
Litter
Mean ± SE
0
1
2
3
4
5
6
7
8
9
10
11
12
13
14
15
16
17
18
19
Experimental week

Figure S1

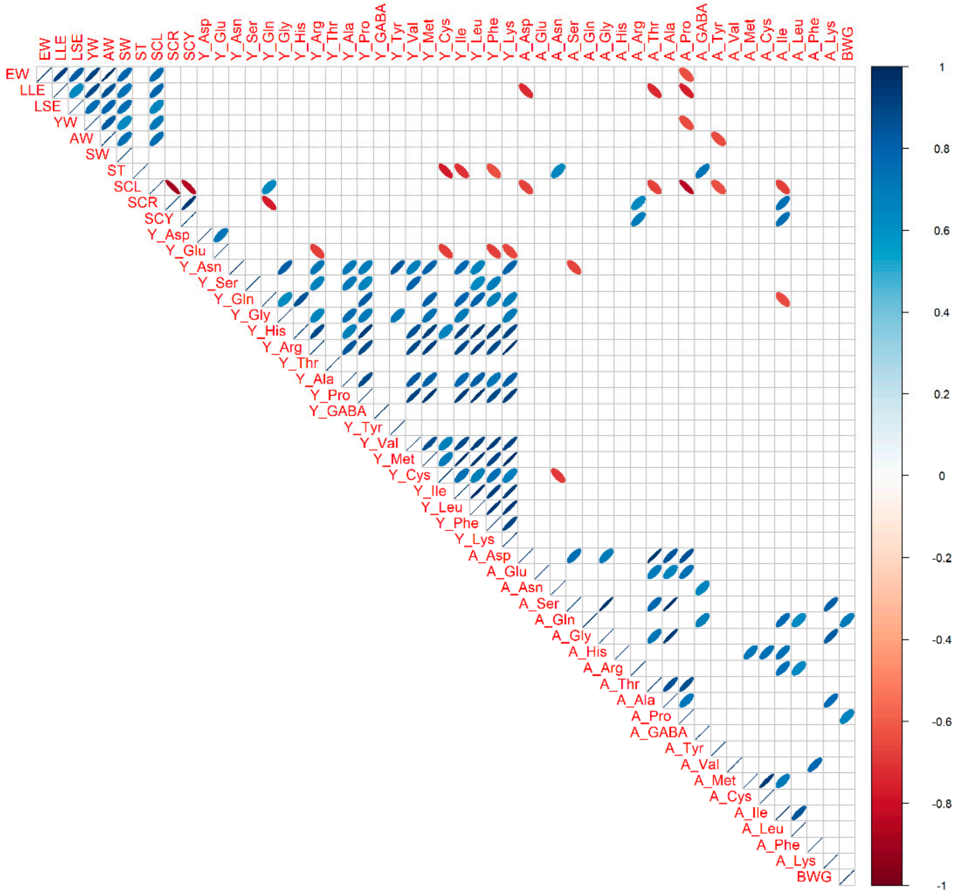
EW
LLE
LSE
YW
AW
SW
ST
SCL
SCR
SCY
Y_Asp
Y_Glu
Y_Asn
Y_Ser
Y_Gln
Y_Gly
Y_His
Y_Arg
Y_Thr
Y_Ala
Y_Pro
Y_GABA
Y_Tyr
Y_Val
Y_Met
Y_Cys
Y_Ile
Y_Leu
Y_Phe
Y_Lys
A_Asp
A_Glu
A_Asn
A_Ser
A_Gln
A_Gly
A_His
A_Arg
A_Thr
A_Ala
A_Pro
A_GABA
A_Tyr
A_Val
A_Met
A_Cys
A_Ile
A_Leu
A_Phe
A_Lys
BWG
1
0.8
0.6
0.4
0.2
0
-0.2
-0.4
-0.6
-0.8
-1

Figure S2

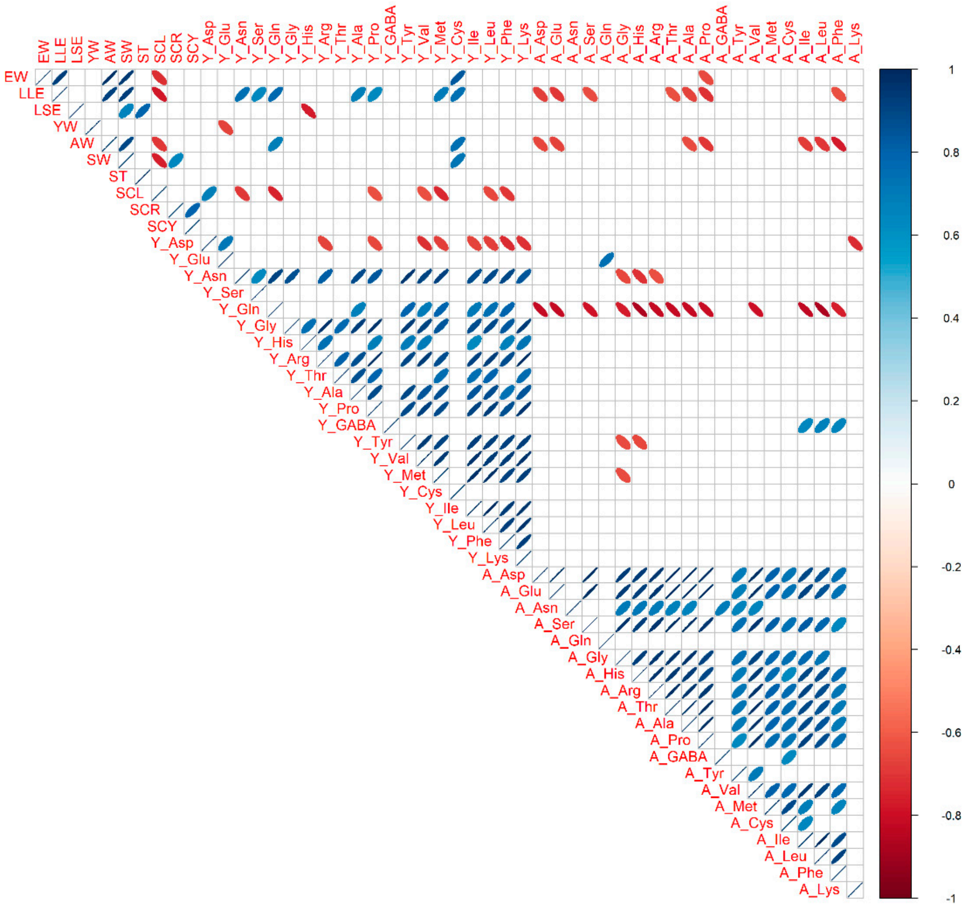
EW
LLE
LSE
YW
AW
SW
ST
SCL
SCR
SCY
Y_Asp
Y_Glu
Y_Asn
Y_Ser
Y_Gln
Y_Gly
Y_His
Y_Arg
Y_Thr
Y_Ala
Y_Pro
Y_GABA
Y_Tyr
Y_Val
Y_Met
Y_Cys
Y_Ile
Y_Leu
Y_Phe
Y_Lys
A_Asp
A_Glu
A_Asn
A_Ser
A_Gln
A_Gly
A_His
A_Arg
A_Thr
A_Ala
A_Pro
A_GABA
A_Tyr
A_Val
A_Met
A_Cys
A_Ile
A_Leu
A_Phe
A_Lys
1
0.8
0.6
0.4
0.2
0
-0.2
-0.4
-0.6
-0.8
-1

Figure S3

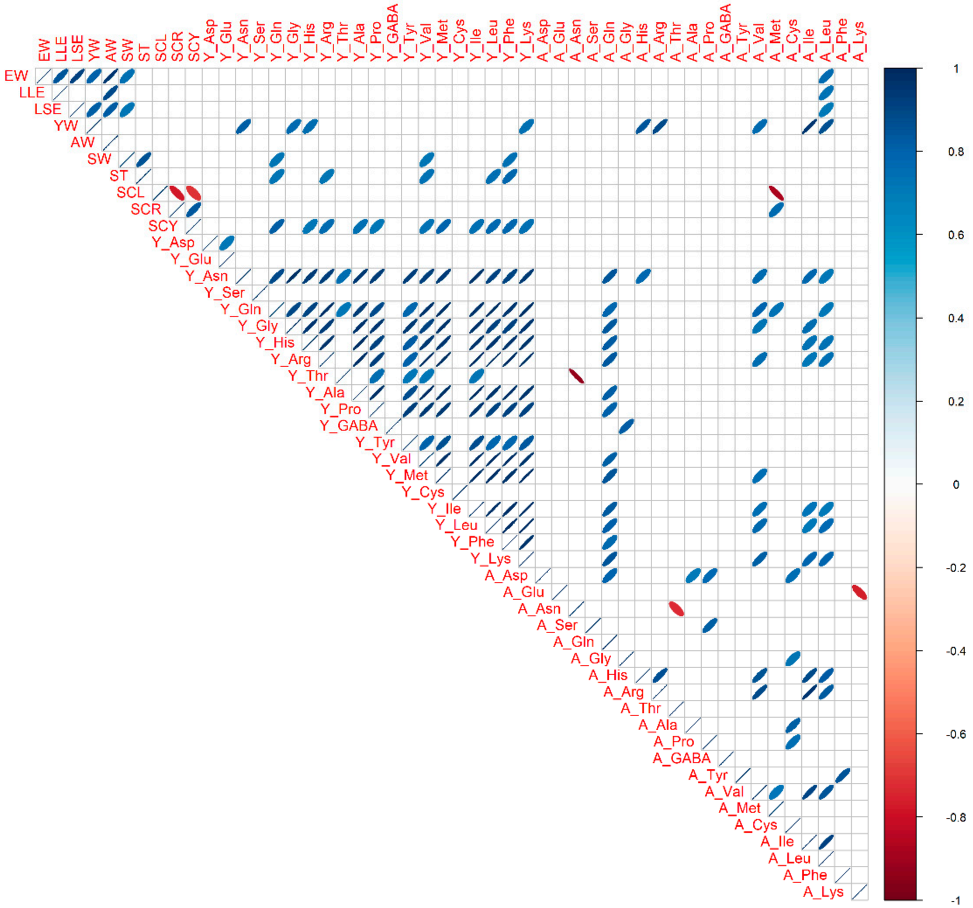
EW
LLE
LSE
YW
AW
SW
ST
SCL
SCR
SCY
Y_Asp
Y_Glu
Y_Asn
Y_Ser
Y_Gln
Y_Gly
Y_His
Y_Arg
Y_Thr
Y_Ala
Y_Pro
Y_GABA
Y_Tyr
Y_Val
Y_Met
Y_Cys
Y_Ile
Y_Leu
Y_Phe
Y_Lys
A_Asp
A_Glu
A_Asn
A_Ser
A_Gln
A_Gly
A_His
A_Arg
A_Thr
A_Ala
A_Pro
A_GABA
A_Tyr
A_Val
A_Met
A_Cys
A_Ile
A_Leu
A_Phe
A_Lys
1
0.8
0.6
0.4
0.2
0
-0.2
-0.4
-0.6
-0.8
-1

Figure S4
